# Cerebral localization of chronic myelomonocytic leukemia: a case report

**DOI:** 10.1186/s13256-021-02947-7

**Published:** 2021-07-25

**Authors:** Clémence Haméon, Cécilia Rousselot, Flavie Arbion, Justine Cibron, Jean-Philippe Cottier, Christophe Destrieux, Olivier Hérault, Emmanuel Gyan

**Affiliations:** 1 Department of Hematology, Tours Hospital, Tours, France; 2 Department of Anatomopathology, Tours Hospital, Tours, France; 3 Department of Radiology, Tours Hospital, Tours, France; 4 Department of Neurosurgery, Tours Hospital, Tours, France; 5 Department of Biology, Tours Hospital, Tours, France

**Keywords:** Chronic myelomonocytic leukemia, Cerebral localization, Case report

## Abstract

**Background:**

Chronic myelomonocytic leukemia is a myelodysplastic/myeloproliferative neoplasm characterized by the infiltration of blood and bone marrow by immature monocytes. Cerebral localization of chronic myelomonocytic leukemia has never been described.

**Case presentation:**

We report the case of a Caucasian 59 year-old man with multiorgan chronic myelomonocytic leukemia infiltration, associated with uncommon brain involvement. There was no evidence of evolution to acute myeloid leukemia. The evidence of cerebral infiltration by chronic myelomonocytic leukemia was made after autopsy.

**Conclusions:**

The fatal outcome of the patient raises the question of the potential benefit of early specific treatment, such as demethylating agents or intensive chemotherapy. Sharing such images of atypical and rapidly evolving chronic myelomonocytic leukemia and the disease history may help clinical decision-making in the future.

## Introduction

Chronic myelomonocytic leukemia (CMML) is a malignant neoplasm that exhibits both myelodysplastic and myeloproliferative features and is characterized by peripheral monocytosis. The clinical presentation is nonspecific, with general symptoms, such as asthenia, night sweats, and weight loss, in symptomatic patients. Neurological manifestations are rare [1].

Molecular biology can detect numerous mutations, such as ASXL1, TET2, and SRSF2. The mutation of SETBP1 is found in 15% of CMML cases [2, 3].

We report the case of a Caucasian 59-year-old man with multiorgan CMML infiltration, including uncommon brain involvement.

## Case presentation

A Caucasian 59-year-old man with a history of hypothyroidy and fatty liver was diagnosed in 2018 with a myelodysplastic syndrome with multilineage dysplasia (MDS-MLD) [4] and a very low risk by the R-IPSS at our facility. There was no family medical history. The patient was married, had two children, and worked as a social worker. He did not consume alcohol nor tobacco. The only treatment was l-thyroxine 50 µg/day.

Laboratory tests showed a leukocyte count of 3.9 × 10^9^L, with neutrophils at 1.5 × 10^9^/L and monocytes at 1.49 × 10^9^/L, a platelet count of 113 × 10^9^/L, and hemoglobin at 115 g/L. The bone-marrow blast count was 1.4%, and cytogenetics were normal, with no Philadelphia chromosome. Next-generation sequencing (NGS) detected a SETBP1 mutation, with a variant allelic fraction (VAF) of 0.21%. Biological and clinical monitoring was decided.

In December 2019, a brutal deterioration of the general state, with a loss of 13 kg, night sweats, major fatigue, and loss of appetite, raised doubts about the diagnosis. The patient’s body temperature was 36.7 °C, heart rate 95 beats per minute, blood pressure 103/64 mmHg, and oxygen saturation at 100% on ambient air. Cardiopulmonary auscultation was normal. He was conscious, oriented, and had a Glasgow score of 15, without detectable abnormalities on neurological clinical examination. His weight was 60.5 kg. Laboratory tests showed a leukocyte count of 29.7 × 10^9^/L, with neutrophils at 23.46 × 10^9^/L and monocytes at 1.78 × 10^9^/L, a platelet count of 83 × 10^9^/L, and hemoglobin at 78 g/L. Another medullary aspiration was performed and showed major dysplasia without excess blasts and a cytological aspect of CMML-0. Immunophenotyping showed a near-absence of type MO3 monocytes, consistent with a diagnosis of CMML. A PET scan was performed and detected several deep lymphadenopathies with moderate hypermetabolism [maximum standardized uptake value (SUV) 3.6]. They had a maximum size of 12 mm and were not puncturable. There was no evidence of another cancer. Treatment with hydroxyurea (three tablets per day) was introduced for the hyperleukocytosis and was administered over 3 weeks.

In January 2020, the appearance of psychomotor slowdown led to admission to the hematology department of Tours Hospital. Subsequently, the patient’s condition quickly worsened with the occurrence of severe problems of alertness and aphasia. At this time, clinical examination showed a Glasgow score of 11 (M6V2E3), with bilateral nonreactive myosis and right central facial paralysis. There was no motor or sensory deficit of the limbs within the limits of clinical examination in such a state of consciousness.

**Fig. 1:**
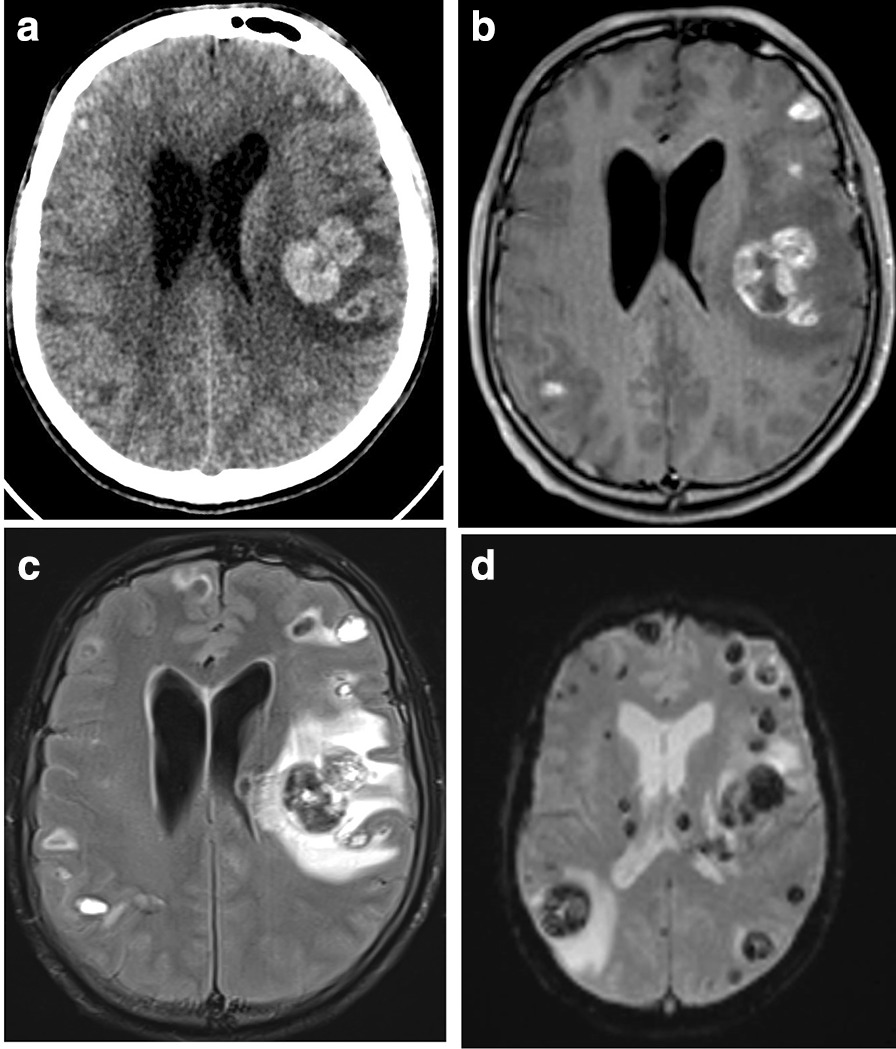
Axial nonenhanced computed tomography (NECT) (**A**) showing multiple hyperdense lesions. The hemorrhagic lesions were very heterogeneous on T1- (**B**), T2-FLAIR (**C**), and perfusion (**D**)-weighted MRI images. The main lesion in the left deep white matter is surrounded by edema, with a mass effect on the ventricle

Laboratory tests showed a hemoglobin level of 77 g/L, a platelet count of 36 × 10^9^/L, and a leukocyte count of 37 × 10^9^/L, with neutrophils at 31 × 10^9^/L and monocytes at 3 × 10^9^/L (Table 1). Infectious assessment was negative, with sterile hemocultures in bacteriology and mycology and negative serologies for *Cryptococcus*, *Toxoplasma*, HIV, HBV, HBC, CMV, EBV, and syphilis. Beta-d-glucan assessment was negative. Autoimmune assessment, including for ANA, was also negative. Cerebral CT showed multiple hemorrhagic nodular lesions (Fig. 1A). On MRI (Fig. 1B–C) these CNS lesions were heterogeneous, without enhancement after gadolinium administration. The MRI also showed multiple supra- and infratentorial hemorrhagic lesions of varying age, in favor of leukemic infiltration as well as the beginning of left temporal engagement (Fig. 1B–D). We could not perform a lumbar puncture because of the risk of engagement. Treatment with high-dose corticosteroids, consisting of 100 mg intravenous methylprednisolone per day, was introduced, and the patient was transferred to intensive care because of his worsening state of consciousness. A neurosurgical diagnosis could not be performed owing to the nontissular hemorrhagic description of the lesions by imaging. The patient died on 15 January 2020.

**Table 1:** Results of laboratory findings on 13 January 2020

Blood counts	
Hemoglobin	77 g/L
Platelets	36 × 10^9^/L
Leukocytes	37 × 10^9^/L
Neutrophils	31 × 10^9^/L
Monocytes	3 × 10^9^/L
Biochemistry	
Sodium	142 mmol/L
Potassium	4.3 mmol/L
Calcium	2.33 mmol/L
Albumin	47 g/L
Phosphates	1.79 mmol/L
Creatinine	97 μmol/L
Glomerular filtration rate (MDRD)	73 mL/min/1.73 m^2^
Uric acid	458 μmol/L
Total bilirubin	10 μmol/L
ASAT	41 UI/L
ALAT	25 UI/L
Alkaline phosphatase	94 UI/L
Gamma-GT	60 UI/L
Lactate dehydrogenase	1039 UI/L
Microbiology	
Hemocultures	Negative
Bacterial	Negative
Fungal	Negative
Urinary analysis	Negative
Beta-d-glucan	Negative
Serologies	
*Cryptococcus*	Negative
*Toxoplasma*	Negative
HIV	Negative
HBV	Negative
HBC	Negative
CMV	Negative
EBV	Negative
Syphilis	Negative

MDRD, modification of diet in renal disease; ASAT, aspartate aminotransferase; ALAT, alanine aminotransferase; HIV, human immunodeficiency viruses; HBV, hepatitis B virus; HBC, hepatitis C virus; CMV, cytomegalovirus; EBV, Epstein–Barr virus

**Fig. 2:**
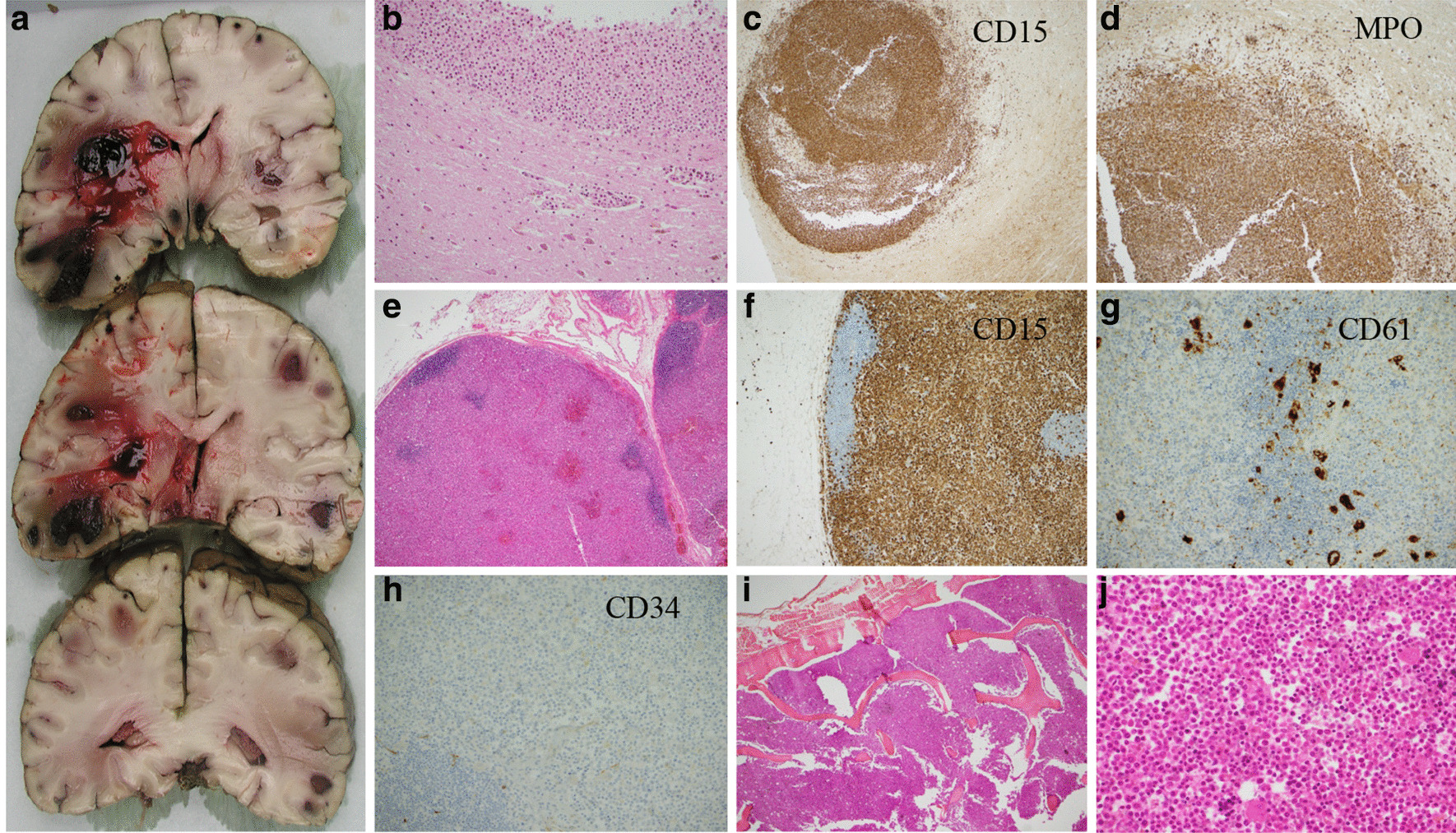
**A** Macroscopic sections of the brain showing the hemorrhagic aspect of the lesions. **B** Microscopic section of the brain with standard hematoxylin and eosin (H&E) coloration; magnification 10×. **C** Microscopic section of the brain with anti-CD15 staining by immunohistochemistry (myelomonocytic marker); magnification 2×. **D** Microscopic section of the brain with anti-MPO staining by immunohistochemistry; magnification 4×. **E** Microscopic section of a lymph node with standard H&E coloration; magnification 2×. **F** Microscopic section of a lymph node with anti-CD15 staining by immunohistochemistry; magnification 4×. **G** Microscopic section of a lymph node with anti-CD61 staining (platelet marker) by immunohistochemistry; magnification 10×. **H** Microscopic section of a lymph node with anti-CD34 staining by immunohistochemistry; magnification 10×. **I** Bone-marrow section; magnification 2×. **J** Bone-marrow section; magnification 20×

An autopsy was performed. Macroscopic sections of the brain showed multiple hemorrhagic lesions (Fig. 2A). Microscopic analyses showed infiltration by relatively immature myeloid elements and hemorrhagic phenomena (Fig. 2B). Immunohistochemistry analyses of the brain tissue showed positivity of the lesion for antibodies against CD15 (Fig. 2C) and MPO (Fig. 2D). No megakaryocytic or erythrocytic precursors were found in the brain (not shown). However, adenopathy (Fig. 2E) exhibited a predominance of myeloid polymorphic localization and mature elements with megakaryocytes (Fig. 2F–G), and elements of erythrocytic lineage (not shown) were also found. No blastic elements were found in the node (Fig. 2H) or other localizations (not shown). The bone marrow (Fig. 2I–J) exhibited a predominance of a myelomonocytic line, but no immature cells. The spleen, lungs, and liver contained mostly myeloid and monocytic elements, without megakaryocytes (not shown). These various elements suggest brain, splenic, liver, lung, and lymph node localization of myelomonocytic leukemia, without any evidence for evolution of the CMML to acute leukemia.

## Discussion and conclusion

We describe the case of a 59 year-old man followed up for 2 years for a CMML who presented with an uncommon presentation of fatal CMML with cerebral and multiorgan involvement. To the best of our knowledge, such diffuse cerebral parenchymal involvement of CMML has not been previously reported in the literature. This case highlights the importance of evoking the diagnosis of cerebral involvement by CMML in the presence of neurologic alterations and multiple MRI brain lesions.

The diagnosis of CMML is made according to the criteria of World Health Organization (WHO) 2016 classification [4] and requires both the presence of persistent monocytosis ≥ 1 × 10^9^/L and monocytes accounting for ≥ 10% of the white blood cell differential count. Two forms of CMML are described according to the WBC count, the “proliferative” type with WBC ≥ 13 × 10^9^/L and the “dysplastic” type with WBC < 13 × 10^9^/L.

The bone marrow blast percentage, which is of prognostic importance, is used to define three groups of CMML: CMML-0, a category for cases with < 2% blasts in peripheral blood and < 5% in bone marrow; CMML-1 for cases with 2–4% blasts in peripheral blood and/or 5–9% in the bone marrow and CMML-2 for cases with 5–19% blasts in peripheral blood and/or 10–19% in bone marrow.

Extramedullary involvement by CMML is uncommon. Other cases of brain involvement in the context of CMML have been reported but are substantially different from ours.

Meningeal infiltration was described by Rogulj *et al*. in which the patient presented with headache, and MRI showed signs of leukoencephalopathy and cortical atrophy. The bone marrow showed CMML in an accelerated phase. The patient was treated by chemotherapy with cytarabine and methotrexate for cerebral diffusion, which relieved the symptoms [5]. In a case of CML in blast crisis, multiple small enhancing nodules in the brain, with myeloid blasts in the central nervous system and diffuse leptomeningeal enhancement during the blast crisis, were described by Lai *et al*. [6]. In our case, signs of leukemic evolution of the CMML were sought and found to be absent, and the MRI imaging characteristics were completely different. In one case of CMML, chronic subdural fluid collection was reported by Bernat *et al*., but the patient presented with a unique lesion that could be drained by craniotomy. The authors also discussed medical treatment and radiation therapy [7]. A brain pseudotumoral inflammatory lesion was described by Joubert *et al*. in 2013 [1]. These cases can all be explained by systemic inflammatory and/or autoimmune manifestations, such as those that occur in connective tissue disease or systemic vasculitis, [8, 9] which may be associated with myeloid malignancies.

Our case underlines the necessity of ruling out differential diagnoses of cerebral pseudoinflammatory tumors, as well as leukemic evolution of CMML and CML. The absence of Philadelphia chromosome and of hyperleukocytosis at diagnosis ruled out the diagnoses of CML, atypical CML (aCML), and chronic neutrophilic leukemia.

There have been no reports of such brain lesions in the differential diagnosis of MDS. Furthermore, lymphadenopathy infiltration with ectopic hematopoiesis has never been described in the context of CMML.

In accordance with the EHA recommendations [10], the treatment of CMML also depends on the risk group.

For low-risk patients, monitoring alone may be sufficient, or in the case of anemia, transfusions support can be provided.

For patients who are not eligible for allogenic transplantation, several therapies exist. Hydroxyurea can be used for proliferative CMML0-1 without significant cytopenia, whereas hypomethylating agents are used in CMML1-2 with significant cytopenia. Allogeneic bone marrow transplantation remains the treatment of choice for younger patients with higher-risk disease, and intensive chemotherapy may be considered as a bridge to transplant in aggressive presentations.

The fatal outcome of our patient raises the question of the potential benefit of early specific treatment, such as demethylating agents or intensive chemotherapy.

This unusual description of a rapidly evolving CMML associated with the multiple-hemorrhage MRI presentation may help clinical decision-making in the future.

## Data Availability

Not applicable.

## Abbreviations

AML: Acute myeloid leukemia
ANA: Antinuclear antibodies
CMML: Chronic myelomonocytic leukemia
CML: Chronic myeloid leukemia
CMV: Cytomegalovirus
CT-Scan: Computed tomography scan
EBV: Epstein–Barr virus
EHA: European Hematology Association
HBV: Hepatitis B virus
HCV: Hepatitis C virus
HIV: Human immunodeficiency viruses
MDS: Myelodysplastic syndrome
MPD: Myeloproliferative disease
MPO: Myeloperoxidase
MRI: Magnetic resonance imaging
NGS: Next-generation sequencing
PET scan: Positron emission tomography scan
R-IPSS: Revised international prognostic scoring system
VAF: Variant allelic fraction
WBC: White blood cells
